# Recrudescent wave of pandemic A/H1N1 influenza in Mexico, winter 2011-2012: Age shift and severity

**DOI:** 10.1371/currents.RRN1306

**Published:** 2012-03-26

**Authors:** Gerardo Chowell, Santiago Echevarría-Zuno, Cecile Viboud, Lone Simonsen, Concepcion Grajales Muñiz, Ramón Alberto Rascón Pacheco, Margot González León, Víctor Hugo Borja Aburto

**Affiliations:** ^*^Dirección de Prestaciones Médicas, Instituto Mexicano del Seguro Social, Mexico City, México; ^†^Fogarty International Center, National Institutes of Health, Bethesda, MD, USA; ^‡^Department of Global Health, George Washington University, School of Public Health and Health Services, DC; ^§^Coordinación de Vigilancia Epidemiológica y Apoyo en Contingencias. Instituto Mexicano del Seguro Social. México City; ^¶^Coordinación de Vigilancia Epidemiológica y Apoyo en Contingencias, Instituto Mexicano del Seguro Social, Mexico City, México and ^**^Coordinación de Vigilancia Epidemiológica y Apoyo en Contingencias, Instituto Mexicano del Seguro Social, México City, México

## Abstract

Background

A substantial recrudescent wave of pandemic influenza A/H1N1 that began in December 2011 is ongoing and has not yet peaked in Mexico, following a 2-year period of sporadic transmission. Mexico previously experienced three pandemic waves of A/H1N1 in 2009, associated with higher excess mortality rates than those reported in other countries, and prompting a large influenza vaccination campaign. Here we describe changes in the epidemiological patterns of the ongoing 4th pandemic wave in 2011-12, relative to the earlier waves in 2009. The analysis is intended to guide public health intervention strategies in near real time.

Methods

We analyzed demographic and geographic data on all hospitalizations with acute respiratory infection (ARI) and laboratory-confirmed A/H1N1 influenza, and inpatient deaths, from a large prospective surveillance system maintained by the Mexican Social Security medical system during 01-April 2009 to 10-Feb 2012. We characterized the age and regional patterns of A/H1N1-positive hospitalizations and inpatient-deaths relative to the 2009 A/H1N1 influenza pandemic. We also estimated the reproduction number (R) based on the growth rate of the daily case incidence by date of symptoms onset.

Results

A total of 5,795 ARI hospitalizations and 186 inpatient-deaths (3.2%) were reported between 01-December 2011 and 10-February 2012 (685 A/H1N1-positive inpatients and 75 A/H1N1-positive deaths). The nationwide peak of daily ARI hospitalizations in early 2012 has already exceeded the peak of ARI hospitalizations observed during the major fall pandemic wave in 2009. The mean age was 34.3 y (SD=21.3) among A/H1N1 inpatients and 43.5 y (SD=21) among A/H1N1 deaths in 2011-12. The proportion of laboratory-confirmed A/H1N1 hospitalizations and deaths was higher among seniors >=60 years of age (Chi-square test P<0.001) and lower among younger age groups (Chi-square test, P<0.03) for the 2011-2012 pandemic wave, compared to the earlier waves in 2009. The reproduction number of the winter 2011-12 wave in central Mexico was estimated at 1.2-1.3, similar to that reported for the fall 2009 wave, but lower than that of spring 2009.

Conclusions

We have documented a substantial and ongoing increase in the number of ARI hospitalizations during the period December 2011-February 2012 and an older age distribution of laboratory-confirmed A/H1N1 influenza hospitalizations and deaths, relative to 2009 A/H1N1 pandemic patterns. The gradual change in the age distribution of A/H1N1 infections in the post-pandemic period is reminiscent of historical pandemics and indicates either a gradual drift in the A/H1N1 virus, and/or a build-up of immunity among younger populations.

## **INTRODUCTION** 

The resurgence of swine-origin pandemic A/H1N1 influenza virus in winter 2011-12 is causing a sizable epidemic in Mexico, following a 2-year period of sporadic transmission. Mexico experienced a series of three A/H1N1 pandemic waves in the spring, summer, and fall of 2009 [1] [2] [3], followed by a large pandemic vaccination campaign towards the end of 2009. These 3 waves were together associated with high excess mortality burden relative to that seen in other countries [4] [5] [6]. Because a significant fraction of the population is now protected from A/H1N1 influenza through natural exposure or vaccination [7], there is potential for the emergence of drift A/H1N1 influenza variants, and/or changing age patterns, as typically seen in post-pandemic  periods [8] [9].  

Here we report on the epidemiology of a recrudescent (4^th^) wave of pandemic A/H1N1 influenza activity in Mexico from 01-December 2011 to 10-February 2012. Because past pandemic experiences have indicated substantial post-pandemic morbidity and mortality burden may occur months to years after the initial pandemic waves [9] [10] [11] [12] [13], we must remain vigilant and continue to monitor the epidemiology and health burden of A/H1N1 influenza. We compared the epidemiological characteristics of laboratory-confirmed A/H1N1 hospitalizations and deaths in winter 2011-12 with those previously reported for the 2009 pandemic waves and show a significant change in the age distribution of cases and deaths.  

## **MATERIALS AND METHODS** 

### **Epidemiological Data**

Individual level hospitalization data were available from a prospective epidemiological surveillance system that was put in place especially for the 2009 influenza pandemic by the Mexican Institute for Social Security (IMSS) [4] [14] [15]. IMSS is a tripartite Mexican health system covering approximately 40% of the Mexican population comprising workers in the private sector and their families, relying on a network of 1,099 primary health-care units and 259 hospitals nationwide. The age and gender distributions of persons affiliated to the IMSS medical system are representative of the general Mexican population [4].   

We analyzed information from all hospitalizations and inpatient-deaths among patients admitted with acute respiratory infection (ARI) during 01-December 2011 to 10-February 2012. ARI was defined as any person with respiratory difficulty presenting fever 38°C and cough together with one or more of the following clinical symptoms: confinement to bed, thoracic pain, polypnea, or acute respiratory distress syndrome. Children <5 years with pneumonia or severe pneumonia that required hospitalization were also considered as ARI cases. Respiratory swabs were obtained for about 26% of ARI hospitalizations (ARI) in winter 2011-12 and were tested for the influenza virus by rRT-PCR [16].

      For all ARI hospitalizations, we retrieved demographic information (age in yrs, and gender), influenza laboratory test result (if tested), reporting state (including 31 states plus the Federal District), and dates of onset of symptoms (self-reported). We also obtained population data by state and age group for all persons affiliated with IMSS in 2009 to calculate incidence rates.    

### **Age distribution and severity of A/H1N1 influenza in 2009 and 2011-12**

We examined the age distribution of hospitalizations and deaths based on all ARIs and laboratory-confirmed A/H1N1 influenza patients reported from 01-December 2011 to 10-February 2012. We compared the age distribution of hospitalizations and deaths in winter 2011-12 with those described for the three waves of the 2009-10 A/H1N1 pandemic in Mexico, 01-April 2009 to 31-March 2010, using the same IMSS reporting system.

We also calculated preliminary estimates of the in-hospital case fatality rate by dividing inpatient deaths by hospitalizations, separately for ARI and laboratory-confirmed A/H1N1. These estimates are preliminary as we likely underestimate the true fatality ratio due to a delay from symptoms onset to death.     

### **Spatial distribution of A/H1N1 influenza in winter 2011-12 and reproduction number estimate**

We analyzed state- and age-specific time series of laboratory-confirmed A/H1N1 influenza hospitalizations by day of symptom onset to analyze the geographic dissemination patterns of ongoing sustained A/H1N1 influenza transmission in Mexico during the early weeks of the wave, 01-December 2011 to 10-February 2012. 

Further, we estimated the reproduction number, R, in Central Mexico where the great majority of cases have been reported, based on a simple method previously used in the context of the 2009 A/H1N1 pandemic waves in Mexico [4]. Specifically we estimate the initial epidemic growth rate by fitting an exponential function to the early ascending phase of daily ARI or A/H1N1 hospitalizations by date of symptoms onset [17]. The early ascending phase was determined as the period between the day of pandemic onset and the midpoint between the onset and peak days. We assumed a mean generation interval of three and four days, which are within the range of mean estimates for the 2009 influenza pandemic [2] [18] [19] [20]. As a sensitivity analysis we also assessed small variations in the length of the ascending epidemic phase used to estimate the exponential growth rate (+/- 4 days). 

This study did not need approval from a scientific committee; all individual data were kept de-identified.

Statistical analyses were performed using SPSS 20.0 and Matlab (The Mathworks, Inc).  

## **RESULTS**

### **Overall epidemiological patterns**

The characteristics of all ARI and A/H1N1-positive hospitalizations reported to the IMSS medical system between 01-Dec 2011 and 10-February 2012 are given in Table 1. The time series of daily ARI hospitalizations and deaths and laboratory-confirmed influenza hospitalizations are shown in Figures 1 and 2, respectively. An A/H1N1  influenza outbreak began around 01-December 2011 and is ongoing at the time of writing of this report (Figure 3), particularly in central Mexico (Figure 1). The daily number of ARI hospitalizations in winter 2011-12 is exceeding the levels that were observed during the major fall wave of the 2009 A/H1N1 influenza pandemic (Figure 4). In Mexico City the cumulative number of ARI hospitalizations during 01-Dec 2011 to 10-February 2012 represents 37% of all ARI hospitalizations that were reported in Mexico City during the first year of A/H1N1 virus circulation (April 2009 to Mar 2010).

**Table:** 

**Variable**	**ARI hospitalizations**	**A/H1N1 confirmed hospitalizations**
**Geographic** Central Southern Other states	2931 (50.6) 992 (17.1) 1872 (32.3)	454 (66.3) 133 (19.4) 98 (14.3)
**Demography** Female	2896 (50)	374 (54.6)
**Age (years)** 0-4 5-14 15-29 30-44 45-59 >=60	1376 (23.8) 497 (8.6) 917 (15.9) 920 (15.9) 837 (14.5) 1236 (21.4)	78 (11.4) 67 (9.8) 169 (24.7) 144 (21) 142 (20.7) 85 (12.4)
**Inpatient severity** Deaths	186 (10.5)	75 (15.6)

**Table 1**. Characteristics of all ARI hospitalizations and laboratory-confirmed A/H1N1 influenza hospitalizations, Mexico, 01 December 2011 through 10 February, 2012.

**Figure:**
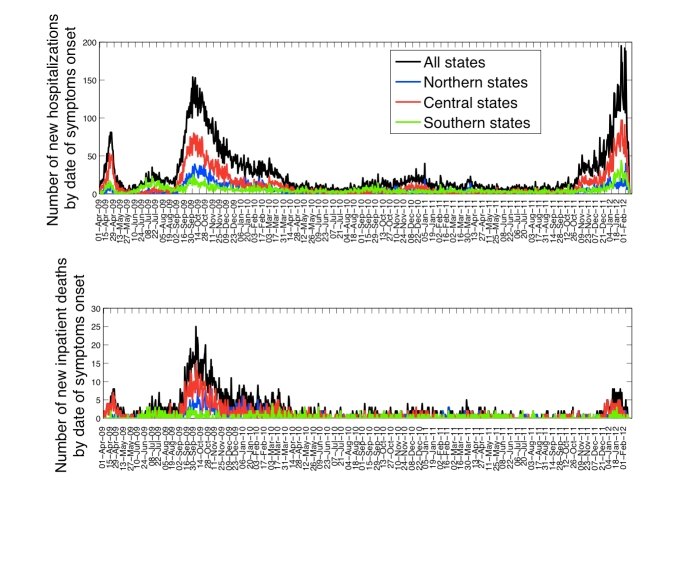
Figure 1: Daily epidemic curves of all ARI hospitalizations (top) and deaths (bottom) by dates of symptoms onset in northern, central, and southeastern states of Mexico, 01-April 2009 to 10-February 2012.

**Figure:**
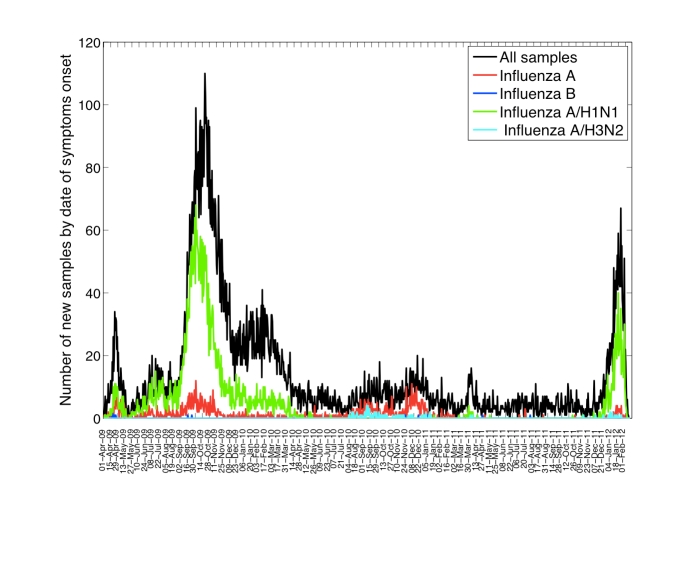
**Figure 2:** Daily number of influenza tests among ARI hospitalizations and laboratory-confirmed influenza hospitalizations by dates of symptoms onset spanning 01-April 2009 to 10-Feberuary 2012 in the 32 Mexican states according to influenza subtype.

**Figure:**
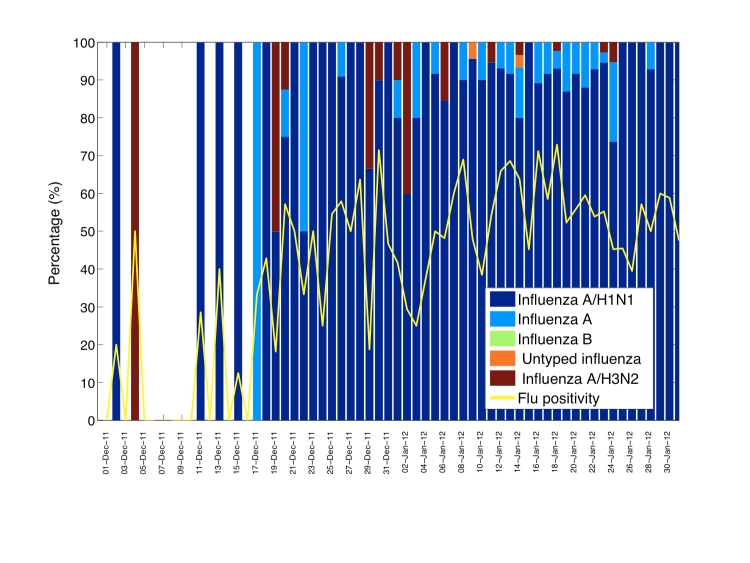
**Figure 3: **Daily influenza positivity rates (no. influenza positive ARI hospitalizations/no. tests among ARI hospitalizations) and percentage of influenza specific subtypes among influenza positive tests from 01-December 2011 to 01-February 2012.

**Figure:**
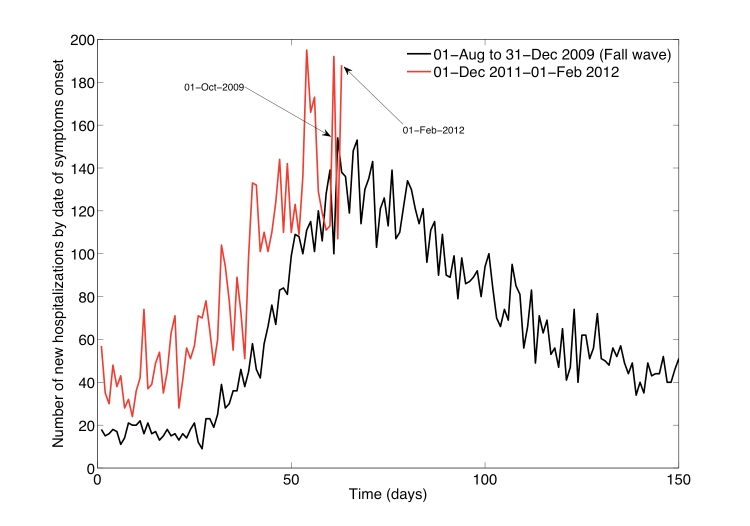
**Figure 4: **Daily epidemic curves of all ARI hospitalizations by dates of symptoms onset across Mexico spanning 01-December to 01-February compared to the fall pandemic wave (Aug-Dec 2009).

### **Severity of disease**

A total of 5,795 ARI hospitalizations and 186 inpatient-ARI deaths (preliminary case fatality rate, 3.2% (95% CI: 2.8, 3.7)) were reported to the IMSS system between 01-December 2011 and 10-February 2012. The preliminary estimate of case fatality rate for laboratory-confirmed A/H1N1 inpatients was 10.9%  (95% CI: 8.6, 13.3) over the same period (685 inpatients and 75 deaths). 

This preliminary estimate of the case fatality rate for hospitalized A/H1N1 patients in 2011-12 is significantly lower than the CFR measured in 2009 (16.1% (95% CI: 15.0, 17.2)).     

### **Age**

Overall the majority of laboratory-confirmed influenza inpatients during 01-Dec 2011 to 10-Feb 2012 were among persons aged 15-59 years (66.4%) followed by the 0-4 year age group (11.4%) and seniors >=60 years (12.4%) (Table 1). Severity increased with older age, with an inpatient fatality rate of 18.8% (95% CI: 10.3, 27.3) for persons >=60 years.   

The cumulative hospitalization and inpatient death rates for the 3 waves of the 2009-10 A/H1N1 pandemic are on average 6.5 and 9.5 times greater than the corresponding rates for the ongoing 2011-12 A/H1N1 wave in Mexico. Comparison of the age-specific A/H1N1 hospitalization and death rates reveals an increasing burden among older populations in 2011-12, relative to the 2009-10 waves (Figure 5). An analysis of the proportionate distribution of A/H1N1 hospitalization and inpatient deaths reveals a shift in the age distribution of recent cases towards older ages as well. Specifically, we note a significantly higher proportion of individuals older than 60 yrs hospitalized with laboratory-confirmed A/H1N1 in 2011-12, relative to the 2009-10 pandemic period (12.4% vs. 6.1%, Chi-square test P<0.0001, Table 2, Figure 6). We also found a reduction in the proportion of A/H1N1-positive hospitalizations among persons 5-14 years of age compared to the 2009 pandemic (9.8% vs. 14.9%, Chi-square test, P=0.0003).

We found a similar change in the age distribution of A/H1N1 inpatient deaths in 2011-12 compared to the 2009 A/H1N1 influenza pandemic (Table 3, Figure 6). Specifically, 21.3% of deaths occurred among persons >=60 years of age in the ongoing 2011-12 epidemic period whereas only 8.9% in the 2009-10 period (Chi-square test, P=0.0006). Similarly to the age shift in hospitalization data, the proportion of A/H1N1 inpatient deaths among individuals aged 15-29 declined, relative to 2009-10 (9.3% vs. 21%, Chi-square test, P=0.02).  

**Figure:**
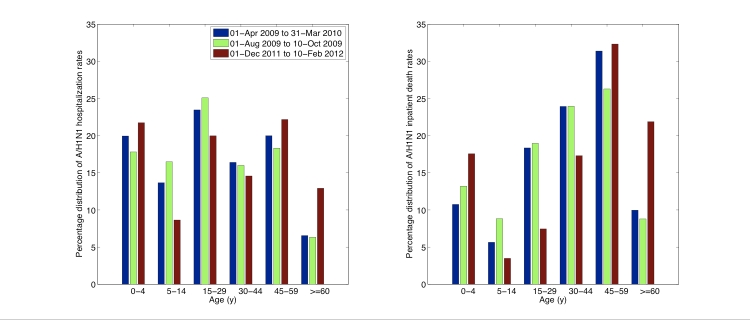
**Figure 5:** Relative distribution of age-specific A/H1N1 influenza hospitalization rates (left) and A/H1N1 inpatient death rates (right) for the ongoing A/H1N1 influenza epidemic (01-Dec 2011 to 10-Feb 2012) compared to those of the entire 2009 A/H1N1 pandemic period (01-Apr 2009 to 31-Mar 2010) and to the first 70 days of the 2009 fall pandemic wave (01-Aug 2009 to 10-Oct 2009). 

**Table:** 

	01-Apr 2009 to 31-Ma 2010		01-Dec 2011 to 10-Feb 2012		
	Total	Proportion of hospitalizations (%)	Total	Proportion of hospitalizations (%)	P value^*^
**Total**	4420	100%	685	100%	
0-4	446	10.1%	78	11.4%	0.30
5-14	660	14.9%	67	9.8%	**0.0003**
15-29	1237	28%	169	24.7%	0.071
30-44	1010	22.9%	144	21.0%	0.29
45-59	798	18.1%	142	20.7%	0.09
>=60	269	6.09%	85	12.4%	**<0.0001**

^*^ **Computed using the Chi-square test statistic for differences in time periods**   **Table 2.** Age-specific proportions of total laboratory-confirmed A/H1N1 hospitalizations for the 2009 A/H1N1 influenza pandemic compared to ongoing A/H1N1 outbreaks in Mexico spanning 01-December 2011 to 10-February 2012. We note a significantly different age distribution of A/H1N1 hospitalizations during 01-December 2011 to 10-February 2012 compared to that of the 2009 A/H1N1 influenza pandemic spanning 01-April 2009 to 31-March 2010 (Wilcoxon test, P<0.0001).  

**Table:** 

	01-Apr 2009 to 31-Mar 2010		01-Dec 2011 to 10-Feb 2012		P value^*^
	Total	Proportion of deaths (%)	Total	Proportion of deaths (%)	
**Total**	711	**100%**	75	**100%**	
0-4	37	5.2%	7	9.3%	0.14
5-14	42	5.91%	3	4%	0.50
15-29	149	21%	7	9.3%	**0.02**
30-44	227	31.9%	19	25.3%	0.24
45-59	193	27.1%	23	30.7%	0.52
>=60	63	8.86%	16	21.3%	**0.0006**

^*^ **Computed using the Chi-square test statistic for differences in time periods**

**Table 3.** Age-specific proportions of total laboratory-confirmed A/H1N1 inpatient deaths for the 2009 A/H1N1 influenza pandemic and ongoing A/H1N1 outbreaks in Mexico spanning 01-December 2011 to 10-February 2012. We note a significantly different age distribution of A/H1N1 inpatient deaths in the 4th wave, compared to that of the previous 3 waves during 2009 (Wilcoxon test, P=0.001).  The age shift is characterized by a doubled proportion of elderly deaths, offset by a halving in deaths in young adults.

**Figure:**
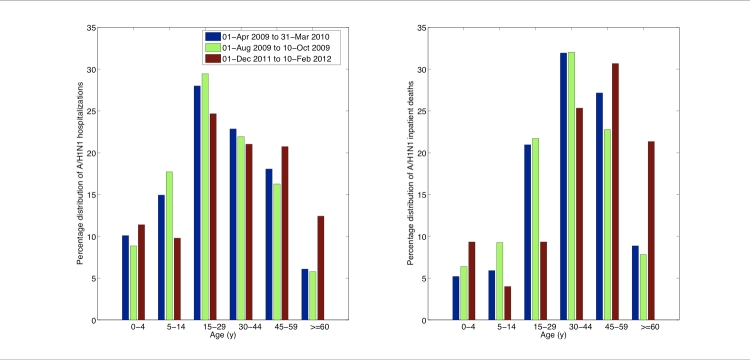
**Figure 6:** Age-specific proportions of A/H1N1 influenza hospitalizations (left) and A/H1N1 inpatient deaths (right) for the ongoing A/H1N1 influenza epidemic (01-Dec 2011 to 10-Feb 2012) compared to those of the entire 2009 A/H1N1 pandemic period (01-Apr 2009 to 31-Mar 2010) and to the first 70 days of the 2009 fall pandemic wave (01-Aug 2009 to 10-Oct 2009).

### **Geography**

The majority of A/H1N1 inpatients during the 4^th^ wave have been reported in central Mexican states (66.3%) followed by southeastern states (19.4%) (Table 1), and a higher proportion of A/H1N1 deaths have occurred in central states compared to other regions (Chi-square test, P=0.04).

### **Estimates of the reproduction number**

Assuming a mean generation interval of 3 (and 4) days, the mean R was estimated to be 1.2 (1.3) during the period 17-Dec 2011 to 9-Jan 2012, based on daily A/H1N1-positive hospitalizations. As a sensitivity analysis we also estimated R using daily ARI hospitalizations; our estimate of R was somewhat lower at 1.1 (1.2). When the length of the epidemic ascending phase was varied (+/- 4 days), our R estimates changed by 0.1 or less.     

## **DISCUSSION**

We have characterized the epidemiology of a recrudescent 4^th^ wave of A/H1N1 influenza transmission in Mexico spanning 01-December 2011 to 10-Feberuary 2012, based hospitalizations for acute respiratory infections and laboratory-confirmed A/H1N1 infections. We compared the impact, severity, age patterns, and reproduction number of this 4th wave with those of earlier pandemic waves in spring, summer and fall 2009 in Mexico, [1] [4]. We used individual-level patient information collected through a prospective influenza surveillance system put in place especially for the 2009 pandemic by the largest Mexican Social Security medical system and providing daily data during 2009-2012 [14] . Our data show that the nationwide peak level of daily ARI hospitalizations obtained so far in early 2012 (it may not yet have peaked) has already exceeded the peak of ARI hospitalizations observed during the major fall pandemic wave in 2009. We have also documented a significant increase in the proportion of A/H1N1 hospitalizations and deaths among persons >=60 y, relative to the 2009 pandemic, and a significant reduction in the proportion of A/H1N1 hospitalizations and deaths among school age children.   

The observed change in age distribution of hospitalization and deaths in the post 2009 pandemic period is reminiscent of the influenza seasons following the 1918 influenza pandemic [8] [9] [21] and the 1968 pandemic [22]. A quantitative analysis of excess mortality prior to and after the1918 influenza pandemic found that the age distribution of influenza-related mortality returned to pre-pandemic mortality levels a few years after the initial pandemic waves as a result of emerging drift variants [9] [23].  Hence the age shift seen in the 2011-12 winter season could signal either a gradual emergence of drift A/H1N1 variants, and/or a build up of immunity among younger populations. Both have implications for influenza prevention and mitigation strategies, which we discuss below.

 During the first year of circulation of the 2009 A/H1N1 influenza pandemic virus, protection from influenza-related morbidity and mortality rates was reported in people over 60 years. This phenomenon of “senior sparing” in age cohorts born prior to the 1957 pandemic is consistent with first exposure to antigenically-related A/H1N1 viruses in childhood, a pattern consistent with the antigen recycling and original antigenic sin hypotheses [1] [24] [25] [26] [27]. A high fraction of the Mexican population is now protected against the 2009 A/H1N1 influenza virus through natural exposure in 2009 (children and young adults) or prior immunity (seniors) [7] and by  pandemic vaccines. Over 7 million of seasonal influenza vaccine (featuring a good match for the H1N1-pdm vaccine component) were administered in 2011-12 winter (35% vaccination coverage among IMSS-affiliated seniors >=60 years; 70% among <5 years; 40% among 50-59 years; and 24% among 5-9 years).

 Although we saw evidence of a shift in the age distribution of  2011-12 cases towards seniors, the absolute risk of getting hospitalized was still relatively low in this age group, relative to those in younger adults. The declining rates of severe cases in younger age groups is most consistent with build-up of immunity. Overall the age distribution of recent A/H1N1 influenza hospitalizations and deaths in Mexico is relatively flat and not quite back to the normal “J-shaped” age risk profile that characterizes seasonal influenza. In the long run, we expect the pandemic A/H1N1 virus to drift genetically to escape mounting population immunity – perhaps with the result that seniors are no longer protected [1]. Hence the epidemiological evidence is consistent with the genetic and antigenic information published on circulating influenza virus, suggesting a lack antigenic drift in A/H1N1 viruses in Mexico or elsewhere in winter 2011-12, a season associated with relatively low A/H1N1 activity globally [28].

Since transmission of the A/H1N1 influenza virus was sporadic in the winter of 2010-2011 in Mexico, we cannot rule out the possibility of some loss of population immunity since 2009. We estimated a reproduction number for the ongoing A/H1N1 epidemic to be significantly lower to that of the spring (R~1.8-2.1) and summer (R~1.6-1.9) pandemic waves in 2009 in Mexico, but in close agreement with estimates of the fall (3rd) 2009 wave (R~ 1.2-1.3)[4].

Perhaps the most surprising finding of this analysis is the occurrence of a substantial 4^th^ wave of pandemic A/H1N1 activity in Mexico, a country which has already experienced severe excess mortality impact during 3 waves of transmission in 2009 [4] [6]. Although we are just beginning to assess the global mortality burden of the 2009 A/H1N1 virus in the pandemic and post-pandemic period, important geographical variations in the number, timing, transmissibility and impact of sequential pandemic waves are obvious. For instance, the UK experienced 2 waves in spring and fall 2009, to be followed by a relatively severe recrudescent wave in 2010-11, not seen in other European countries [29]. The US experienced the brunt of the pandemic burden in the first year of A/H1N1 circulation. To our knowledge, the 4^-^wave pattern seen in Mexico in 2009-12 has not been reported in other countries. Whether these differences can be explained by geographical variation in prior immunity, seasonal drivers, control strategies, connectivity, health and healthcare, is unclear and remains a key area for future research.

In summary our findings indicate a changing age distribution of laboratory-confirmed A/H1N1 influenza hospitalizations and deaths in winter 2011-12, relative to 2009-10 A/H1N1 pandemic patterns. The proportion of hospitalizations and deaths is increasing in seniors >=60 years, an age group that was largely protected during the early pandemic waves in 2009. In contrast, rates of A/H1N1 hospitalizations and deaths are declining among younger population groups, consistent with a gradual build up of immunity. This gradual change in the age distribution A/H1N1 influenza in 2011-12 in Mexico is reminiscent of post-pandemic patterns in past influenza pandemic. As the 4^th^ wave is still ongoing, it is too early to determine whether it is more severe than the previous waves in terms of mortality – something that occurred in the 1889 pandemic in which a 3^rd^ wave occurring in the winter of 1891-92 was far more deadly than previous waves [13] [30].

Whether other countries will eventually experience similar severe recrudescent waves of A/H1N1 activity remains to be seen. A multinational comparison of the epidemiology of pandemic and post-pandemic waves would be useful to shed light on the long-term transmission dynamics and build up of immunity to pandemic viruses, and inform control strategies.  

## **COMPETING INTERESTS**

The authors declare no relevant competing interests.    

## **FUNDING INFORMATION**

This research was conducted in the context of the MISMS (Multinational Influenza Seasonal Mortality Study), an ongoing international collaborative effort to understand influenza epidemiological and evolutionary patterns, led by the Fogarty International Center, National Institutes of Health (http://www.origem.info/misms/index.php). The MISMS study is funded by the International Influenza Unit, Office of Global Health Affairs, Department of Health and Human Services. LS acknowledges support from the RAPIDD (Research and Policy for Infectious Disease Dynamics) program of the Science and Technology Directorate, Department of Homeland Security, and the Fogarty International Center.

## **CORRESPONDING AUTHOR**

Dr. Víctor H. Borja-Aburto  Coordinación de Vigilancia Epidemiológica y Apoyo en Contingencias,  Instituto Mexicano del Seguro Social,  Mier y Pesado 120, México, DF 03100 México  Email: victor.borja@imss.gob.mx

## References

[ref-1455513854] Chowell G, Viboud C, Simonsen L, Miller MA, Acuna-Soto R, Díaz JM, Martínez-Martín AF. The 1918-19 influenza pandemic in boyacá, Colombia. Emerg Infect Dis. 2012 Jan;18(1):48-56. doi: 10.3201/eid1801.101969. 22257780 10.3201/eid1801.101969 PMC3310082

[ref-2775801215] Fraser C, Donnelly CA, Cauchemez S, Hanage WP, Van Kerkhove MD, Hollingsworth TD, Griffin J, Baggaley RF, Jenkins HE, Lyons EJ, Jombart T, Hinsley WR, Grassly NC, Balloux F, Ghani AC, Ferguson NM, Rambaut A, Pybus OG, Lopez-Gatell H, Alpuche-Aranda CM, Chapela IB, Zavala EP, Guevara DM, Checchi F, Garcia E, Hugonnet S, Roth C; WHO Rapid Pandemic Assessment Collaboration. Pandemic potential of a strain of influenza A (H1N1): early findings. Science. 2009 Jun 19;324(5934):1557-61. Epub 2009 May 11. 19433588 10.1126/science.1176062 PMC3735127

[ref-3172867402] Perez-Padilla R, de la Rosa-Zamboni D, Ponce de Leon S, Hernandez M, Quiñones-Falconi F, Bautista E, Ramirez-Venegas A, Rojas-Serrano J, Ormsby CE, Corrales A, Higuera A, Mondragon E, Cordova-Villalobos JA; INER Working Group on Influenza. Pneumonia and respiratory failure from swine-origin influenza A (H1N1) in Mexico. N Engl J Med. 2009 Aug 13;361(7):680-9. Epub 2009 Jun 29. 19564631 10.1056/NEJMoa0904252

[ref-2600093662] Chowell G, Echevarría-Zuno S, Viboud C, Simonsen L, Tamerius J, Miller MA, Borja-Aburto VH. Characterizing the epidemiology of the 2009 influenza A/H1N1 pandemic in Mexico. PLoS Med. 2011 May;8(5):e1000436. Epub 2011 May 24. 21629683 10.1371/journal.pmed.1000436 PMC3101203

[ref-53091159] Zepeda-Lopez HM, Perea-Araujo L, Miliar-García A, Dominguez-López A, Xoconostle-Cázarez B, Lara-Padilla E, Ramírez Hernandez JA, Sevilla-Reyes E, Orozco ME, Ahued-Ortega A, Villaseñor-Ruiz I, Garcia-Cavazos RJ, Teran LM. Inside the outbreak of the 2009 influenza A (H1N1)v virus in Mexico. PLoS One. 2010 Oct 8;5(10):e13256. 20949040 10.1371/journal.pone.0013256 PMC2951908

[ref-1300595585] Charu V, Chowell G, Palacio Mejia LS, Echevarría-Zuno S, Borja-Aburto VH, Simonsen L, Miller MA, Viboud C. Mortality burden of the A/H1N1 pandemic in Mexico: a comparison of deaths and years of life lost to seasonal influenza. Clin Infect Dis. 2011 Nov;53(10):985-93. Epub 2011 Oct 5. 21976464 10.1093/cid/cir644 PMC3202315

[ref-550782419] Elizondo-Montemayor L, Alvarez MM, Hernández-Torre M, Ugalde-Casas PA, Lam-Franco L, Bustamante-Careaga H, Castilleja-Leal F, Contreras-Castillo J, Moreno-Sánchez H, Tamargo-Barrera D, López-Pacheco F, Freiden PJ, Schultz-Cherry S. Seroprevalence of antibodies to influenza A/H1N1/2009 among transmission risk groups after the second wave in Mexico, by a virus-free ELISA method. Int J Infect Dis. 2011 Nov;15(11):e781-6. Epub 2011 Aug 19. 21855383 10.1016/j.ijid.2011.07.002 PMC4041370

[ref-3130320126] Simonsen L, Clarke MJ, Schonberger LB, Arden NH, Cox NJ, Fukuda K. Pandemic versus epidemic influenza mortality: a pattern of changing age distribution. J Infect Dis. 1998 Jul;178(1):53-60. 9652423 10.1086/515616

[ref-3605372020] Saglanmak N, Andreasen V, Simonsen L, Mølbak K, Miller MA, Viboud C. Gradual changes in the age distribution of excess deaths in the years following the 1918 influenza pandemic in Copenhagen: using epidemiological evidence to detect antigenic drift. Vaccine. 2011 Jul 22;29 Suppl 2:B42-8. 21757103 10.1016/j.vaccine.2011.02.065 PMC3144399

[ref-4123282907] Olson DR, Simonsen L, Edelson PJ, Morse SS. Epidemiological evidence of an early wave of the 1918 influenza pandemic in New York City. Proc Natl Acad Sci U S A. 2005 Aug 2;102(31):11059-63. Epub 2005 Jul 26. 16046546 10.1073/pnas.0408290102 PMC1182402

[ref-533424257] Chowell G, Viboud C, Simonsen L, Miller MA, Acuna-Soto R. Mortality patterns associated with the 1918 influenza pandemic in Mexico: evidence for a spring herald wave and lack of preexisting immunity in older populations. J Infect Dis. 2010 Aug 15;202(4):567-75. PubMed Central PMCID: PMC2945372. 20594109 10.1086/654897 PMC2945372

[ref-2001487809] Chowell G, Viboud C, Simonsen L, Miller MA, Hurtado J, Soto G, Vargas R, Guzman MA, Ulloa M, Munayco CV. The 1918-1920 influenza pandemic in Peru. Vaccine. 2011 Jul 22;29 Suppl 2:B21-6. 21757099 10.1016/j.vaccine.2011.02.048 PMC3144394

[ref-3386203433] Valleron AJ, Cori A, Valtat S, Meurisse S, Carrat F, Boëlle PY. Transmissibility and geographic spread of the 1889 influenza pandemic. Proc Natl Acad Sci U S A. 2010 May 11;107(19):8778-81. Epub 2010 Apr 26. 20421481 10.1073/pnas.1000886107 PMC2889325

[ref-3294099397] Echevarría-Zuno S, Mejía-Aranguré JM, Mar-Obeso AJ, Grajales-Muñiz C, Robles-Pérez E, González-León M, Ortega-Alvarez MC, Gonzalez-Bonilla C, Rascón-Pacheco RA, Borja-Aburto VH. Infection and death from influenza A H1N1 virus in Mexico: a retrospective analysis. Lancet. 2009 Dec 19;374(9707):2072-9. Epub 2009 Nov 11. 19913290 10.1016/S0140-6736(09)61638-X

[ref-2299140976] Chowell G, Viboud C, Simonsen L, Miller M, Echevarría-Zuno S, et al. (2011) Impact of antiviral treatment and hospital admission delay on severity of 2009 A/H1N1 pandemic influenza in Mexico, April-December 2009. In revision. 10.1186/1471-2334-12-97 PMC3449201 22520743

[ref-2619267309] (2009) Centers for Disease Control and Prevention. Serum cross-reactive antibody response to a novel influenza A (H1N1) virus after vaccination with seasonal influenza vaccine. MMWR Morb Mortal Wkly Rep 58: 521-524. 19478718

[ref-2691902701] Wallinga J, Lipsitch M. How generation intervals shape the relationship between growth rates and reproductive numbers. Proc Biol Sci. 2007 Feb 22;274(1609):599-604. PubMed Central PMCID: PMC1766383. 17476782 10.1098/rspb.2006.3754 PMC1766383

[ref-153562134] Yang Y, Sugimoto JD, Halloran ME, Basta NE, Chao DL, Matrajt L, Potter G, Kenah E, Longini IM Jr. The transmissibility and control of pandemic influenza A (H1N1) virus. Science. 2009 Oct 30;326(5953):729-33. Epub 2009 Sep 10. 19745114 10.1126/science.1177373 PMC2880578

[ref-1512363216] Cauchemez S, Donnelly CA, Reed C, Ghani AC, Fraser C, Kent CK, Finelli L, Ferguson NM. Household transmission of 2009 pandemic influenza A (H1N1) virus in the United States. N Engl J Med. 2009 Dec 31;361(27):2619-27. 20042753 10.1056/NEJMoa0905498 PMC3840270

[ref-3445095673] Cowling BJ, Chan KH, Fang VJ, Lau LL, So HC, Fung RO, Ma ES, Kwong AS, Chan CW, Tsui WW, Ngai HY, Chu DW, Lee PW, Chiu MC, Leung GM, Peiris JS. Comparative epidemiology of pandemic and seasonal influenza A in households. N Engl J Med. 2010 Jun 10;362(23):2175-84. 20558368 10.1056/NEJMoa0911530 PMC4070281

[ref-2080726857] Simonsen L, Reichert TA, Miller M (2004) The Virtues of antigenic sin: consequences of pandemic recycling on influenza-associated mortality: Options for the control of influenza V: International Congress Series.

[ref-59550665] Viboud C, Grais RF, Lafont BA, Miller MA, Simonsen L; Multinational Influenza Seasonal Mortality Study Group. Multinational impact of the 1968 Hong Kong influenza pandemic: evidence for a smoldering pandemic. J Infect Dis. 2005 Jul 15;192(2):233-48. Epub 2005 Jun 15. 15962218 10.1086/431150

[ref-2842064138] Andreasen V, Viboud C, Simonsen L. Epidemiologic characterization of the 1918 influenza pandemic summer wave in Copenhagen: implications for pandemic control strategies. J Infect Dis. 2008 Jan 15;197(2):270-8. 18194088 10.1086/524065 PMC2674012

[ref-4008191435] La Ruche G, Tarantola A, Barboza P, Vaillant L, Gueguen J, Gastellu-Etchegorry M; epidemic intelligence team at InVS. The 2009 pandemic H1N1 influenza and indigenous populations of the Americas and the Pacific. Euro Surveill. 2009 Oct 22;14(42). pii: 19366. 19883543 10.2807/ese.14.42.19366-en

[ref-834740084] Viboud C, Miller M, Olson D, Osterholm M, Simonsen L. Preliminary Estimates of Mortality and Years of Life Lost Associated with the 2009 A/H1N1 Pandemic in the US and Comparison with Past Influenza Seasons. PLoS Curr. 2010 Mar 20:RRN1153. PMC2843747 20352125 10.1371/currents.RRN1153 PMC2843747

[ref-3750433735] Miller MA, Viboud C, Balinska M, Simonsen L. The signature features of influenza pandemics--implications for policy. N Engl J Med. 2009 Jun 18;360(25):2595-8. Epub 2009 May 7. 19423872 10.1056/NEJMp0903906

[ref-1523641851] Reichert T, Chowell G, Nishiura H, Christensen RA, McCullers JA. Does Glycosylation as a modifier of Original Antigenic Sin explain the case age distribution and unusual toxicity in pandemic novel H1N1 influenza? BMC Infect Dis. 2010 Jan 7;10:5. 20059763 10.1186/1471-2334-10-5 PMC3003248

[ref-12216603] Centers for Disease Control and Prevention. FluView. 2011-2012 Influenza season Week 5 ending February 4, 2012. Website:

[ref-628377111] Ellis J, Galiano M, Pebody R, Lackenby A, Thompson C, Bermingham A, McLean E, Zhao H, Bolotin S, Dar O, Watson JM, Zambon M. Virological analysis of fatal influenza cases in the United Kingdom during the early wave of influenza in winter 2010/11. Euro Surveill. 2011 Jan 6;16(1). pii: 19760. 21223836

[ref-1299575866] Stuart-Harris CH. Pandemic influenza: an unresolved problem in prevention. J Infect Dis. 1970 Jul-Aug;122(1):108-15. 4914941 10.1093/infdis/122.1-2.108

